# Utility of GATA3, Uroplakin-II, and p40 Markers in the Diagnosis of Urothelial Carcinoma

**DOI:** 10.7759/cureus.113714

**Published:** 2026-07-31

**Authors:** Lavanya Manjari, Amrutha M R, Ramesh K R, Vivek P O, Shruthi H

**Affiliations:** 1 Pathology, Sri Siddhartha Medical College, Sri Siddhartha Academy of Higher Education, Tumkur, IND; 2 Urology, District Hospital, Tumkur, IND

**Keywords:** bladder biopsy, bladder cancer, gata3, immunohistochemistry, p40, transurethral resection of bladder tumor (turbt), uroplakin ii, urothelial carcinoma

## Abstract

Background, aim, and objectives: Accurate diagnosis of urothelial carcinoma (UC), particularly in high-grade and limited biopsy specimens, may be challenging on morphology alone. Immunohistochemistry (IHC) markers such as GATA3, uroplakin II (UPII), and p40 may improve diagnostic confidence. This study aimed to evaluate the diagnostic utility of GATA3, UPII, and p40, individually and as a combined panel, in urothelial carcinoma, and to correlate marker expression with tumor grade and invasion status.

Methods: This cross-sectional study was conducted in the Department of Pathology, Sri Siddhartha Medical College (SSMC), Tumkur, Karnataka, India, over a period of 24 months from February 2024 to January 2026. The study included 35 consecutive histopathologically confirmed cases of urothelial carcinoma received in the Department of Pathology during the study period, diagnosed on transurethral resection of bladder tumor (TURBT) specimens and bladder biopsy specimens. Formalin-fixed paraffin-embedded tissue sections were stained with hematoxylin and eosin and subjected to IHC for GATA3, UPII, and p40. Positivity rates and staining intensity were assessed. Associations with grade and invasion were analyzed statistically. Data were entered into Microsoft Excel and analyzed using SPSS Statistics (IBM Corp., Armonk, NY, USA). Categorical variables were expressed as frequencies and percentages, while continuous variables were summarized as mean ± standard deviation. Associations between marker expression, tumor grade, and invasion status were analyzed using the chi-square test.

Results: The mean age of the study population was 67.09 ± 5.77 years, with a male predominance (25/35, 71.4%). Low-grade urothelial carcinomas constituted 18/35 (51.4%) cases, while 17/35 (48.6%) were high-grade. Positive IHC expression was observed in 34/35 (97.1%) cases for UPII, 33/35 (94.3%) for GATA3, and 32/35 (91.4%) for p40. No statistically significant association was found between the expression of any marker and tumor grade or invasion status (p > 0.05). Triple-marker positivity was observed in 30/35 (85.7%) cases, dual-marker positivity in 4/35 (11.4%), and single-marker positivity in 1/35 (2.9%). No case was negative for all three markers, resulting in 100% diagnostic coverage with the combined IHC panel.

Conclusion: GATA3, UPII, and p40 are highly useful IHC markers in urothelial carcinoma. While each marker performed well individually, the combined panel demonstrated positivity in all cases in this cohort, suggesting complementary diagnostic value. This three-marker panel may be useful in difficult primary and metastatic cases requiring confirmation of urothelial differentiation.

## Introduction

Bladder carcinoma is a malignant tumor of the urinary bladder, with urothelial carcinoma (UC) accounting for nearly 90% of cases. It represents the most common malignancy of the urinary tract [1,2]. In India, bladder cancer ranks among the leading cancers in men, showing a marked male predominance (male:female ratio ~8.6:1), higher than the global average. According to Global Cancer Observatory (GLOBOCAN) 2022, UC ranks 17th in incidence and 19th in mortality in India, with an estimated five-year prevalence of approximately four per 100,000 population and around 13,000 deaths annually [1]. Established risk factors include increasing age, male gender, tobacco smoking, and occupational exposure to carcinogens.

Accurate diagnosis of UC can be challenging, particularly in high-grade, poorly differentiated tumors and limited biopsy samples, where morphological features may overlap with other malignancies such as squamous cell carcinoma and adenocarcinoma. In such settings, immunohistochemistry (IHC) plays a crucial role. Nuclear transcription factor GATA3 is widely used in identifying urothelial differentiation [3]. Uroplakin II (UPII), a transmembrane protein, is relatively specific for urothelial cells and may show reduced expression in high-grade tumors [4,5], while p40, a non-transactivating isoform of p63, is a nuclear marker associated with basal and squamous differentiation and is frequently expressed in UC [6].

However, reliance on a single marker may be limited by variable sensitivity and specificity. Therefore, a panel-based approach is increasingly recommended to improve diagnostic accuracy [7]. Recent studies have also highlighted the importance of molecular heterogeneity and biomarker expression patterns in improving diagnostic precision and subclassification of bladder cancer [8,9]. Furthermore, advances in molecular pathology have enhanced our understanding of bladder cancer biology and have contributed to improved diagnostic and therapeutic strategies [10]. This study aims to evaluate the diagnostic utility of GATA3, UPII, and p40 individually and in combination and to analyze their expression patterns in relation to tumor grade and invasion status in urothelial carcinoma.

## Materials and methods

Study design and setting

This cross-sectional study was conducted by the Department of Pathology, Sri Siddhartha Medical College (SSMC), Tumkur, Karnataka, India, and was approved by the Institutional Ethics Committee (approval no. SSMC/MED/IEC-058/Feb-2024). The study was carried out over a period of 24 months from February 2024 to January 2026. The study included 35 consecutive cases of histopathologically confirmed UC diagnosed on transurethral resection of bladder tumor (TURBT) specimens and bladder biopsies. Cases with inadequate tissue, poorly preserved paraffin blocks, necrosed or autolyzed tissue, and patients with a history of neoadjuvant therapy were excluded from the study.

Specimens were received in 10% formalin and subjected to routine gross examination. Tissue samples were processed, paraffin-embedded, and sectioned at 4 µm thickness. Hematoxylin and eosin (H&E)-stained sections were evaluated histopathologically, and tumors were graded according to standard WHO/International Society of Urological Pathology (ISUP) criteria. Additional 4 µm sections were prepared on positively charged slides for IHC analysis using antibodies against GATA3, UPII, and p40. 

Immunohistochemical expression was assessed semi-quantitatively based on staining intensity and the percentage of positive tumor cells. Both GATA3 and p40 demonstrated nuclear staining, whereas UPII showed membranous and cytoplasmic staining. Each slide was initially scanned at low magnification to evaluate overall staining intensity and distribution.

Statistical analysis was performed using SPSS Statistics version 30.0 (IBM Corp., Armonk, NY, USA) software. Categorical variables were expressed as frequencies and percentages. Associations between marker expression, tumor grade, and invasion status were analyzed using the chi-square test or Fisher’s exact test wherever appropriate. A p-value of less than 0.05 was considered statistically significant.

The sample size was calculated using the formula for estimating a single proportion. Based on a previous study that reported p40 immunoreactivity in 87% of UC cases, an expected proportion (p) of 0.87 was considered. Assuming a 90% confidence level (Z = 1.64) and an absolute precision of 10% (d = 0.10), the minimum required sample size was calculated to be 30 cases. After accounting for a 10% non-response rate, the final sample size was increased to 35 cases. 

## Results

A total of 100% (n=35) cases of histologically confirmed UC were included in the study. The mean age of patients was 67.09 ± 5.77 years, with a clear male predominance (71.4%). Based on histological grading, 18 cases (51.4%) were classified as low-grade UC, while 17 cases (48.6%) were high-grade tumors.

IHC expression

Immunohistochemical analysis demonstrated high overall positivity for all three markers. Uroplakin II showed the highest expression, with positivity in 34 out of 35 cases (97.1%), followed by GATA3 in 33 cases (94.3%) and p40 in 32 cases (91.4%). Both GATA3 and p40 exhibited predominantly strong nuclear staining, whereas UPII showed membranous and cytoplasmic staining with variable intensity.

Correlation with tumor grade and invasion

The association between IHC marker expression and tumor grade was assessed using Fisher’s exact test. No statistically significant association was observed between marker expression and tumor grade for GATA3 (p = 1.000), p40 (p = 1.000), or UPII (p = 1.000). Similarly, the relationship between marker expression and invasion status was evaluated using Fisher’s exact test, which showed no significant association for GATA3 (p = 0.441), p40 (p = 0.208), or UPII (p = 0.977). The association between staining intensity and tumor category was analyzed using the chi-square test, which did not demonstrate statistical significance (χ² = 15.91, p = 0.069). However, a trend toward stronger staining intensity in invasive high-grade urothelial carcinomas was observed.

Combined marker panel analysis

Evaluation of the combined IHC panel demonstrated triple-marker positivity in 30 cases (85.7%), dual-marker positivity in four cases (11.4%), and single-marker positivity in one case (2.9%). Notably, none of the cases were negative for all three markers, resulting in 100% diagnostic coverage when the panel was used in combination. The occasional negativity of individual marker combinations may reflect tumor heterogeneity and loss of antigen expression in poorly differentiated UCs. The complementary use of all three markers overcame this limitation and achieved complete diagnostic coverage in the present cohort (Tables 1-2).

**Table 1:** Distribution of tumor category and histological grade in UC UC: Urothelial carcinoma

Category	Cases (n)	Percentage
Invasive: High grade	12	34.3
Invasive: Low grade	6	17.1
Non-invasive: High grade	5	14.3
Non-invasive: Low grade	12	34.3
Total	35	100.0

**Table 2:** Diagnostic utility of selected IHC marker combinations UPII: Uroplakin II, IHC: Immunohistochemistry

Marker combination	Cases (n)	Percentage	Sensitivity (%)
GATA3 + UPII	34	97.1	97.1
GATA3 + p40	34	97.1	97.1
GATA3 + UPII + p40	35	100.0	100.0

Staining intensity patterns

**Figure 1:**
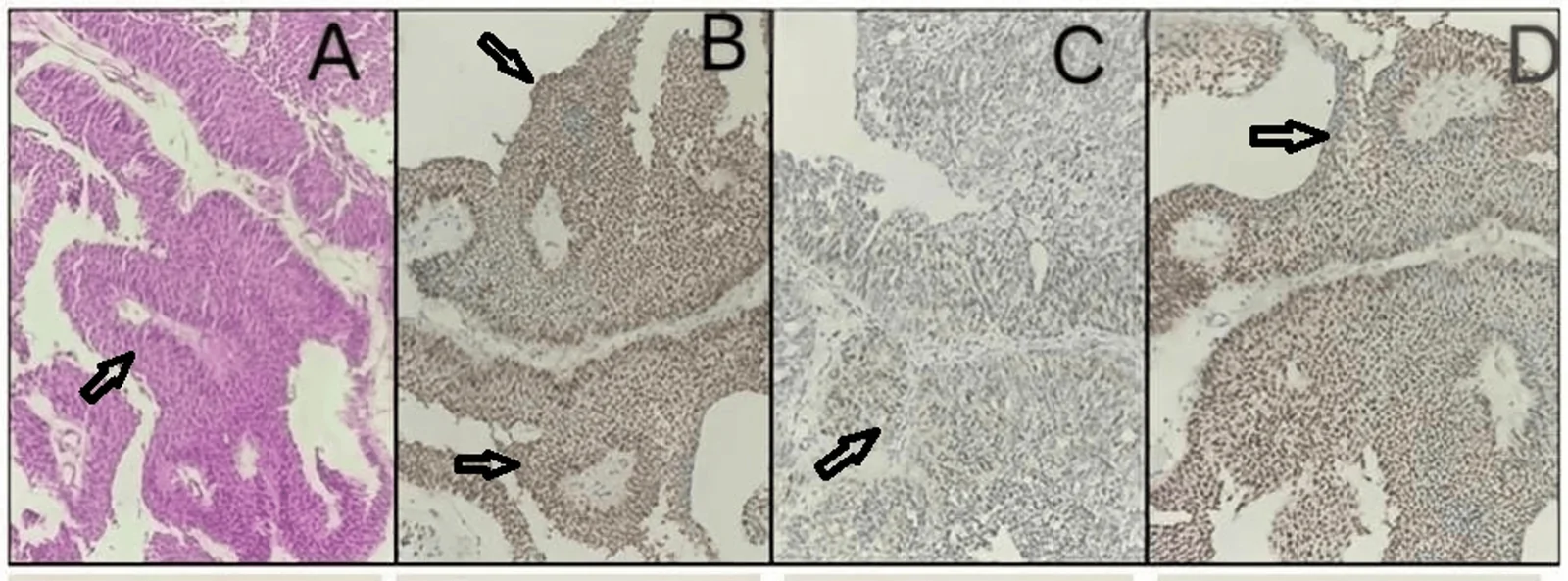
Non-invasive UC (200×) A: Hematoxylin and eosin (H&E, 200×). The arrow indicates papillary fronds lined by atypical urothelial cells without stromal invasion, consistent with non-invasive papillary UC. B: GATA3 IHC (200×). The arrows indicate diffuse strong nuclear positivity in the neoplastic urothelial cells. C: UPII IHC (200×). The arrow points to weak membranous/cytoplasmic staining in the tumor cells. D: p40 IHC (200×). The arrow shows strong diffuse nuclear positivity in the neoplastic urothelial cells. UC: Urothelial carcinoma, IHC: Immunohistochemistry, UP II: Uroplakin II

**Figure 2:**
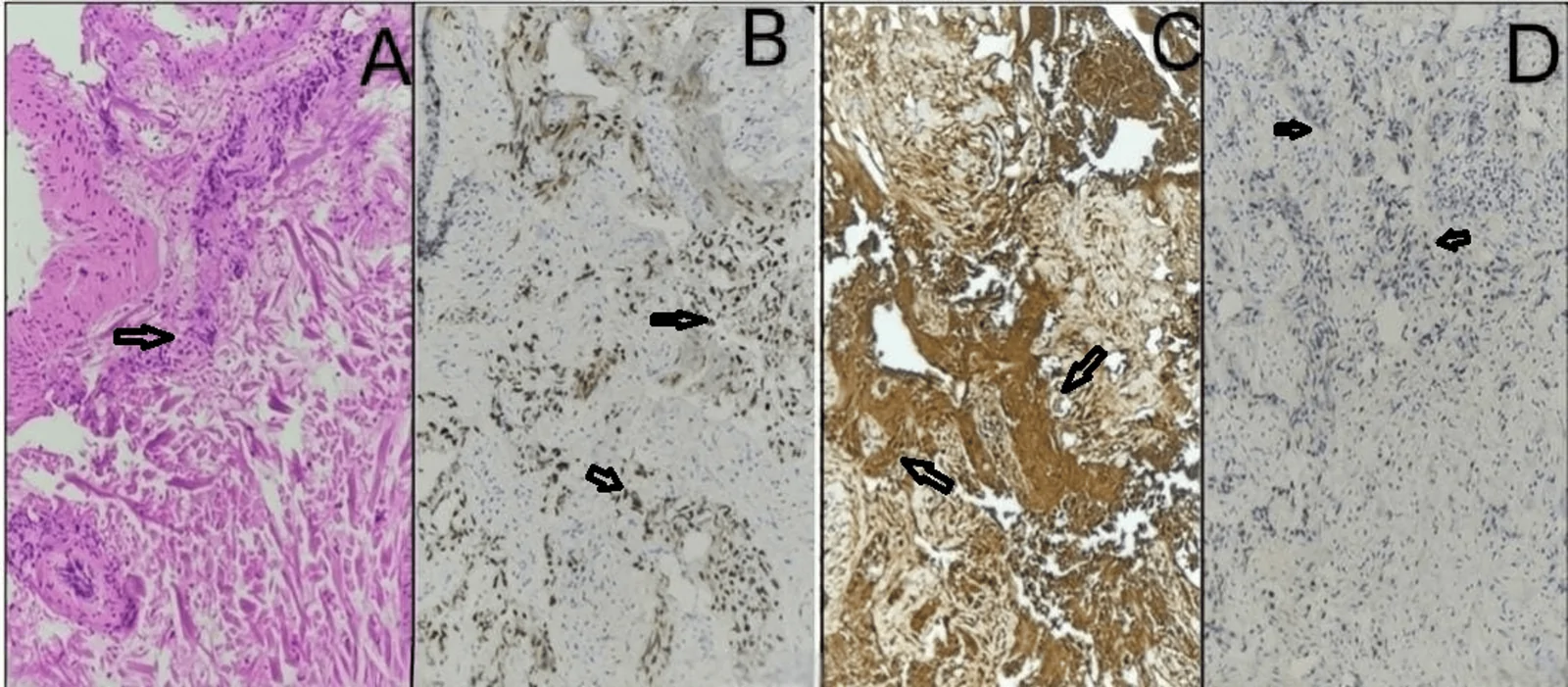
Invasive UC (200×) A: H&E (200×). The arrow indicates infiltrating nests of malignant urothelial cells invading the fibrous stroma, consistent with invasive UC. B: GATA3 IHC (200×). The arrows point at tumor cell nuclei showing moderate nuclear positivity, confirming urothelial differentiation. C: UPII IHC (200×). The arrows indicate strong diffuse membranous/cytoplasmic positivity in invasive tumor cells, supporting urothelial origin. D: p40 IHC (200×). The arrows highlight scattered tumor cell nuclei showing weak nuclear positivity within the invasive carcinoma. UC: Urothelial carcinoma, H&E: Hematoxylin and eosin, IHC: Immunohistochemistry, UP II: Uroplakin II

Assessment of staining intensity revealed that GATA3 and p40 predominantly showed moderate to strong nuclear expression across both low-grade and high-grade tumors. Uroplakin II demonstrated variable intensity, with relatively reduced expression in some high-grade lesions, suggesting loss of differentiation in more aggressive tumors (Figures 1-2).

## Discussion

Urothelial carcinoma is the most common malignancy of the urinary bladder and continues to present diagnostic challenges, particularly in high-grade tumors, variant histology, and limited biopsy specimens. In such settings, IHC serves as a critical adjunct to morphology. The present study evaluates the diagnostic utility of GATA3, UPII, and p40, individually and as a combined panel, and correlates their expression with tumor grade and invasion status.

Clinicopathological profile

The mean age of patients in this study was 67.09 ± 5.77 years, with a male predominance (71.4%), consistent with global epidemiological trends reported in large population-based studies such as GLOBOCAN 2022 and other institutional series [1,10,11]. The nearly equal distribution of low-grade and high-grade tumors in this cohort reflects a representative pathological spectrum and is comparable to findings reported by Amin et al. and Warrick et al. [2,8].

GATA3 expression

The GATA3 is a well-established marker of urothelial differentiation with consistently high sensitivity across multiple studies [12,13]. In the present study, GATA3 showed positivity in 94.3% of cases (Table 1), comparable to previous reports by Palicelli et al., Sanfrancesco et al., and Miettinen et al. [3,11,14]. Although reduced expression has been reported in poorly differentiated and high-grade tumors because of loss of differentiation and intratumoral heterogeneity [8], no significant association with tumor grade was observed in the present study, supporting its role as a reliable diagnostic marker. 

p40 expression

The p40 demonstrated positivity in 91.4% of cases (Table 1), consistent with previous studies [6]. Although p40 is not entirely specific for urothelial differentiation, it is useful in identifying basal and squamous differentiation and serves as a complementary marker when interpreted with GATA3 and UPII.

UPII expression

Uroplakin II demonstrated the highest positivity rate (97.1%) in the present study (Table 1), in agreement with Hoang et al. and other published studies reporting high sensitivity and specificity for urothelial differentiation [15]. Although reduced expression has been described in poorly differentiated tumors, overall positivity remained high in our cohort, supporting its value as a diagnostic marker.

Correlation with tumor grade and invasion

The present study found no statistically significant association between the expression of GATA3, UPII, or p40 and tumor grade or invasion status. Similar observations have been reported in prior studies, indicating that these markers primarily serve a diagnostic rather than a prognostic role [3,8]. While some studies have suggested reduced expression of differentiation markers in high-grade tumors, such variations were not statistically significant in our cohort, possibly due to sample size limitations.

Combined marker panel utility

Triple-marker positivity was observed in 85.7% of cases, while no case was negative for all three markers, indicating complete diagnostic coverage within this cohort. Occasional loss of individual marker expression may reflect tumor heterogeneity or reduced antigen expression in poorly differentiated tumors. These findings support the complementary use of GATA3, UPII, and p40, particularly in diagnostically challenging cases. Similar panel-based approaches have been recommended in previous studies to improve diagnostic confidence [3,7,8,13].

Comparison with existing literature

Overall, the findings of the present study are consistent with published literature demonstrating high expression of GATA3, p40, and UPII in UC. Minor differences in positivity rates among studies are likely related to differences in sample size, antibody clones, scoring criteria, and tumor heterogeneity (Table 3).

**Table 3:** Comparative analysis of demographic profile, histological grade distribution, and IHC expression of GATA3, p40, and UPII in UC across published studies and the present study The present study demonstrated high positivity for GATA3 (94.3%), p40 (85.7%), and UPII (97.1%). The observed expression rates were comparable to those reported in previous studies, particularly Leivo et al. [7] and Abdulkhaliq et al. [15]. The combined marker panel provided complete diagnostic coverage, supporting the utility of a multimarker IHC approach in urothelial carcinoma. UC: Urothelial carcinoma, IHC: Immunohistochemistry, UPII: Uroplakin II

Authors and referenced study	Year	Sample size (n)	Male (%)	Female (%)	M:F ratio	GATA3 positivity (%)	p40 positivity (%)	UPII positivity (%)
Lin et al. [6]	2014	72	NR	NR	NR	83–90	94	NR
Hoang et al. [5]	2015	63	NR	NR	NR	86	85	77
Leivo et al. [7]	2016	89	78	22	3.5:1	99	87	80
Agarwal et al. [16]	2019	74	85	15	5.7:1	77	NR	NR
Salama and Khairy [13]	2022	60	NR	NR	NR	93.3	NR	86.7*
Abdulkhaliq and Jalal [15]	2024	79	NR	NR	NR	94.9	NR	NR
Present Study	2026	35	71.4	28.6	2.5:1	94.3 (33/35)	85.7 (30/35)	97.1 (34/35)

Clinical implications

The combined GATA3-UPII-p40 panel may be particularly useful in evaluating poorly differentiated tumors, limited biopsy specimens, tumors with divergent differentiation, and metastatic carcinomas of unknown primary origin. Accurate confirmation of urothelial differentiation can facilitate appropriate diagnosis and clinical management [17-20].

Limitations

The study is limited by its relatively small sample size and single-center design. Additionally, the absence of a control group comprising non-urothelial tumors limits assessment of specificity. Long-term follow-up data were not available to evaluate prognostic significance.

Future directions

Further multicentric studies with larger cohorts and inclusion of histological mimics are recommended to validate these findings. Evaluation of additional markers and molecular subtyping may further enhance diagnostic accuracy and provide prognostic insights.

## Conclusions

The present study demonstrates that the combined IHC panel of GATA3, UPII, and p40 provides comprehensive diagnostic coverage for confirming urothelial differentiation in UC. No triple-negative cases were identified in this cohort, highlighting the value of this panel in diagnostically challenging settings, including high-grade tumors, poorly differentiated neoplasms, limited biopsy specimens, and metastatic tumors of uncertain primary origin. Among the individual markers, UPII showed the highest sensitivity, followed closely by GATA3 and p40.

Although the expression of GATA3, UPII, and p40 was not significantly associated with tumor grade or invasion status, their consistently high positivity supports their role as diagnostic rather than prognostic biomarkers. These findings support the use of this three-marker panel as a useful adjunct to routine histopathological evaluation of UC. Multicenter studies with larger sample sizes are warranted to validate these findings and further establish the diagnostic utility of this IHC panel in routine practice.
